# Obituary

**Published:** 1889-07

**Authors:** A. W. Clement, Wm. Dougherty, C. K. Dyer

**Affiliations:** Committee; Committee; Committee


### OBITUARY.

CHARLES B. MOULTON, M. D., D. V. S., died the latter part of March in Washington. Dr. Moulton was born in Manchester, New Hampshire. Was graduated at the American Veterinary College in 1882, entered the U. S. Army as a veterinarian and served some time on the frontier. He was afterwards transferred to Washington and promoted to be Chief Veterinary Surgeon in the Quartermaster's department. While stationed at Washington he pursued a course of medical study in the University of Georgetown where he graduated as an M. D. in 1887. Very soon after this he was transferred to St. Louis, where he remained until the year 1888, when he resigned his position in the army and returned to Washington to engage in private practice. The following resolutions were passed by the Maryland State Veterinary Society.

WHEREAS It has pleased Almighty God, in His wisdom, to remove from our midst our friend and professional brother, Charles B. Moulton, M.D., D.V.S., of Washington, D. C., who was one of the founders of this society, and who had ever taken an active interest in all that concerns the welfare of the veterinary profession :

*Resolved*, That we, the Maryland State Veterinary Society, deeply deplore his loss to the veterinary profession and especially to this society, and that we extend our heartfelt sympathy to his family in their bereavement.

*Resolved*, That these resolutions be spread upon the minutes of this society and published in the *American Veterinary Review*, the JOURNAL OF COMPARATIVE MEDICINE AND SURGERY, and the *Baltimore Sun*.

*Resolved*. That a copy of these resolutions be sent to the family of the deceased.

A. W. CLEMENT,
WM. DOUGHERTY,
C. K. DYER,
*Committee.*

Dr. Ezra Mink, V.S., died March 25, in Rochester, aged 65. He was born in this State and for the past sixteen years has practiced in the above city. Resolutions of condolence were passed by the Rochester Veterinary Medical Association.

